# Critical dialogue: Can AI-based imaging predict tumor biology? Infer metastasis, microvascular invasion, and growth patterns from pixels

**DOI:** 10.1186/s13244-025-02136-w

**Published:** 2025-11-10

**Authors:** Yashbir Singh, Gregory J. Gores, Jesper B. Andersen, Sara Salehi, Bradley J. Erickson

**Affiliations:** 1 Radiology, Mayo Clinic, Rochester, MN USA; 2 Division of Gastroenterology & Hepatology, Mayo Clinic, Rochester, MN USA; 3 Biotech Research and Innovation Centre (BRIC), Department of Health and Medical Sciences, University of Copenhagen, Copenhagen, Denmark

## Current practice and knowledge

Traditional cancer staging and treatment planning rely greatly on morphological imaging characteristics, histopathological analysis, and molecular biomarkers obtained through invasive procedures [1]. Current radiological assessments focus primarily on tumor size, anatomical location, and gross morphological features to guide clinical decision-making. However, these conventional approaches provide limited insight into the underlying tumor biology that drives aggressive behavior, metastatic potential, and treatment resistance. Existing imaging modalities, including CT, MRI, and positron emission tomography (PET), capture structural and functional information, yet the interpretation remains largely qualitative and dependent on the expertise of the individual radiologist [2]. While contrast enhancement patterns and apparent diffusion coefficients provide some biological insights, the correlation between imaging features and molecular characteristics remains inadequately explored. A fundamental limitation of current imaging-based approaches is the inherent resolution difference between imaging pixels and cellular-level histological structures. Medical imaging pixels represent tissue volumes significantly larger than individual cells or microscopic architectural features that define tumor biology. This scale disparity means that imaging-based predictions rely on statistical correlations between macroscopic imaging patterns and underlying microscopic biological processes, rather than direct visualization of cellular or molecular characteristics.

Current practice often requires tissue sampling for definitive characterization of tumor aggressiveness, microvascular invasion (MVI), and molecular subtyping, which carries inherent risks and sampling limitations.

The concept of AI-derived imaging biomarkers, linking imaging phenotypes to molecular signatures, has emerged as a promising bridge between non-invasive imaging and biological understanding [3]. However, implementation in routine clinical practice remains limited due to the complexity of correlating high-dimensional imaging data with molecular profiles, limited data, and the need for robust validation across diverse patient populations and institutions.

## Advancements and new developments

Recent developments in AI, particularly deep learning and machine learning algorithms, have revolutionized the potential for extracting quantitative biological information from medical images [4]. Today, advanced AI models are capable of analyzing thousands of imaging features that are invisible to the human eye, identifying subtle patterns that correlate with tumor biology and clinical outcomes. Convolutional neural networks (CNNs) have demonstrated remarkable success in predicting molecular subtypes [5], gene expression profiles [6, 7], and protein markers directly from imaging data across multiple cancer types [8, 9]. Studies have shown that AI models capable of predicting ER (Estrogen Receptor), PR (Progesterone Receptor), and HER2 (Human Epidermal Growth Factor Receptor 2) status in breast cancer from MRI with accuracy comparable to immunohistochemistry.

However, while these results are promising in breast imaging, the translation to other cancer types faces significant challenges. The expanding repertoire of histological and molecular markers across different malignancies, particularly the growing number of targetable biomarkers for precision therapies, presents increasing complexity for AI-based prediction models. As new therapeutic targets emerge and histopathological classification systems evolve, the ability of imaging-based AI to keep pace with this molecular diversity remains to be extensively validated across cancer types beyond the well-studied breast and lung cancer applications.

Similarly, AI algorithms have been used to successfully identify microsatellite instability (MSI) status in colorectal cancer [10] and predict EGFR (Epidermal Growth Factor Receptor) mutations in lung cancer from routine imaging studies [11].

Recent AI breakthroughs include the development of models that can predict MVI in hepatocellular carcinoma from pre-operative imaging with promising accuracy, serving as a valuable supportive tool for surgical planning decisions while complementing rather than replacing pathological evaluation [12]. Multi-modal AI approaches combining imaging with clinical data have achieved remarkable performance in predicting metastatic potential, with some models demonstrating the ability to identify high-risk patients, who would benefit from more aggressive surveillance or adjuvant therapy [13].

Radiomics powered by machine learning has revealed imaging signatures associated with tumor hypoxia, angiogenesis, and immune infiltration [14]. These AI-derived biomarkers correlate strongly with treatment response, achieving area under the curve (AUC) values of 0.84–0.89 for treatment response prediction [15] and demonstrate significant survival outcomes with hazard ratios ranging from 1.5 to 6.2 for overall survival prediction [16], offering unprecedented insights into characteristics of the tumor microenvironment that influence therapeutic efficacy.

## Considerations for radiology practice

The integration of AI-based biological inference into radiology practice represents a paradigm shift toward precision oncology [17].

It is crucial to recognize that AI-based imaging serves as a predictive and supportive tool rather than a replacement for traditional pathological examination. While AI algorithms can identify statistical associations between imaging patterns and biological outcomes, they operate within inherent accuracy limitations due to the scale differences between imaging pixels and cellular structures. These AI-derived predictions should be integrated into clinical decision-making as complementary biomarkers that enhance rather than substitute conventional diagnostic approaches.

Radiologists must adapt to incorporating quantitative AI-derived biomarkers alongside traditional morphological assessment, requiring new competencies in understanding AI model outputs as well as error and failure modes.

Current AI models demonstrate varying performance across different imaging protocols, scanner manufacturers, and patient populations, necessitating careful validation before clinical implementation. Standardization of imaging acquisition protocols becomes critical to ensure AI model reliability and reproducibility across institutions. The challenge of algorithm generalizability across diverse healthcare systems, implementation of electronic health records, and patient demographics requires ongoing attention and validation studies.

A critical consideration for AI implementation is the disparity in access to advanced AI technologies across healthcare systems. The potential applications of AI-based tumor biology prediction are significantly influenced by institutional resources, including computational infrastructure, technical expertise, and financial capabilities. Rural and resource-limited healthcare settings may face substantial barriers to adopting these technologies, potentially exacerbating healthcare disparities. The requirement for high-quality imaging equipment, robust data management systems, and specialized training creates additional access challenges that must be addressed to ensure equitable implementation of AI-driven precision oncology approaches.

Integration of AI models into existing radiology workflows demands consideration of computational infrastructure, data security, and regulatory compliance [18]. Furthermore, disparities in AI access across healthcare systems may limit the widespread application of these technologies, with resource constraints potentially restricting implementation to well-funded academic centers and large healthcare networks.

The black-box nature of many deep learning models raises questions about interpretability and clinical acceptance, driving development toward explainable AI approaches that provide insight into decision-making processes. Training programs must evolve to prepare radiologists for this AI-augmented future, emphasizing understanding of AI capabilities, limitations, and appropriate clinical application. The potential for AI to predict treatment response and guide therapy selection positions radiologists as key players in multidisciplinary oncology teams, expanding their role beyond diagnosis to treatment planning and patient monitoring, and surveillance.

Quality assurance protocols will have to be developed to monitor the AI model's performance over time and detect potential drift or degradation in accuracy. The rapid pace of AI advancement requires lifelong learning and adaptation within radiology departments to maintain optimal patient care standards.

Future directions include the development of real-time AI analysis during image acquisition, enabling adaptation and execution of optimized imaging protocols, immediate biological characterization, and potentially guiding biopsy procedures or treatment decisions. The integration of AI-derived biological markers with existing clinical decision support systems promises to enhance precision medicine approaches and improve patient outcomes.
